# Synergistic anticancer mechanisms of curcumol and paclitaxel in triple-negative breast cancer treatment may involve down-regulating ZBTB7A expression via the NF-B signaling pathway

**DOI:** 10.22038/IJBMS.2022.64571.14218

**Published:** 2022-05

**Authors:** Anyun Mao, Qinghong Qin, Weiping Yang, Changyuan Wei

**Affiliations:** 1 Department of Thyroid and Breast Surgery, Guangdong Medical University Affiliated Houjie Hospital, Dongguan 523945, People’s Republic of China; 2 Department of Breast Surgery, Guangxi Medical University Cancer Hospital, Nanning 530021, People’s Republic of China

**Keywords:** Curcumol, NF-Ƙb, Paclitaxel, TNBC, ZBTB7A

## Abstract

**Objective(s):**

This study aimed to verify whether curcumol combined with paclitaxel exerted synergistic antiproliferative and proapoptotic effects in MDA-MB-231 mammary cancer cells.

**Materials and Methods:**

The effects of different concentrations of CC, PTX, and their combination on the proliferation of MDA-MB-231 mammary cancer cells were determined by CCK-8 laboratory tests. Combination index (CI) was calculated using CompuSyn software. Colony formation assays, Hoechst 33258 immunofluorescence staining, and flow cytometry were carried out to observe proliferation and apoptosis in each group. The protein expression of PCNA, Bcl-2, Bax, ZBTB7A, p-p65, and NF-ƙB p65 was detected by western blotting. The xenograft tumor volume and body mass of nude mice were measured. Immunohistochemistry was used to detect the expression of PCNA , NF-B p65 and ZBTB7A. TUNEL and DAPI staining were used to detect the apoptosis of tumor cells.

**Results:**

Curcumol combined with paclitaxel exerted a significant inhibitory effect on proliferation of MDA-MB-231 cells in the CCK-8 laboratory test. Hoechst 33258 immunofluorescence staining, flow cytometry, TUNEL, and DAPI apoptosis staining demonstrated that cell apoptosis was the highest in the CC+PTX group *in vivo* and *in vitro*. Expression of PCNA, Bcl-2, ZBTB7A, p-p65, and NF-B p65 was lowest in the CC+PTX group, while the expression of Bax was highest. The growth of xenograft tumors in the CC+PTX group was most notably suppressed. Immunohistochemistry showed that expression of PCNA, ZBTB7A, and NF-ƙB p65 was the lowest in the CC+PTX group.

**Conclusion:**

Curcumol combined with paclitaxel exerted a synergistic antiproliferative and proapoptotic effect on triple-negative breast cancer cells

## Introduction

The incidence and mortality of mammary cancer rank first among female malignancies worldwide (1). Triple-negative breast cancer (TNBC) is generally recognized as a unique molecular subtype of mammary cancer. In 2006, Bryan et al. clearly defined TNBC as mammary cancer characterized by lack of expression of estrogen receptor (ER), progesterone receptor (PR), and human epidermal growth factor receptor 2 (HER-2) (2). Given molecular typing characteristics, the efficacy of endocrine therapy and anti-HER-2 targeted therapy against TNBC is poor. In recent years, although new molecular therapies targeting breast cancer susceptibility gene 1 (BRCA1), vascular endothelial growth factor receptor (VEGFR), and PI3K/AKT/mTOR, as well as immunotherapy and androgen receptor inhibitor bicalutamide, have made some progress in improving the treatment efficacy of TNBC, the combination chemotherapy of taxane and anthracycline remains the standard chemotherapy regimen (3-5).

Curcumol (CC) is a natural sesquiterpenoid. Several studies have confirmed that CC exerts significant anti-inflammatory, antiviral, antioxidant, and antifibrotic effects (6). In terms of antitumor effects, CC, which causes low toxicity, exerts antiproliferative and proapoptotic effects on nasopharyngeal carcinoma, gastric cancer, cervical cancer, and lung cancer (7). By reducing the expression of the target gene CDKL3 and arresting the cell cycle in the G1 phase, CC inhibits proliferation and promotes apoptosis in cholangiocarcinoma HCCC-9810 and RBE cells (8).

A prominent hallmark of malignant tumors is the unrestricted proliferation and inhibited apoptosis of tumor cells. PCNA, Bax, and Bcl-2 have been confirmed by a large number of studies to be important proliferation and apoptosis proteins that can reflect the anti-tumor effect of drugs in breast cancer. Studies have confirmed that the NF-κB signal transduction pathway is directly or indirectly involved in inflammatory responses, cell proliferation, apoptosis, angiogenesis, immunosuppression, therapy resistance, and metabolic processes (9, 10). Huang Y et al. found that the NF-κB signaling pathway participated in the paclitaxel-induced apoptosis of mammary cancer, ovarian cancer, and epidermoid carcinoma cells (11). Studies have shown that monomers of natural medicines can inhibit proliferation and promote apoptosis of tumor cells by modulating the expression of NF-κB p65, which is one of the members of the NF-κB family (12, 13). ZBTB7A (zinc finger and BTB domain-containing protein 7A) is a member of the POK (POZ and Krüppel) transcriptional repressor family. ZBTB7A has been considered to be a proto-oncogene closely related to the occurrence and development of mammary cancer. Inhibition of MCF-7 cell proliferation was significantly associated with knockdown of ZBTB7A expression (14). The expression of ZBTB7A was reported to be positively correlated with tumor size, lymphatic metastasis, and survivin expression (15).

Previous studies have confirmed that chemotherapy drugs combined with monomers of natural medicines can more effectively inhibit tumor cell proliferation, promote apoptosis, inhibit metastasis, and improve efficacy (16, 17). Therefore, we chose CC and paclitaxel (PTX) to treat MDA-MB-231 cells to determine whether the combination drugs could exert a synergistic antiproliferative and proapoptotic effect. In addition, we want to explore the underlying mechanism.

## Materials and Methods

***Cell culture and reagents***

MDA-MB-231 cells were purchased from the Cell Bank of the Chinese Academy of Sciences (Shanghai, China). The cells were cultured in Dulbecco’s modified Eagle medium supplemented with 10% fetal bovine serum (BI, USA), 1% penicillin, and streptomycin. The standard culture atmosphere in the humidified incubator was maintained at 37 ^°^C with 5% CO_2_. CC powder (purity≥98%) and PTX for injection were procured from the National Institute for the Control of Pharmaceutical and Biological Products (Beijing, China) and Yangtze River Pharmaceutical Group (Jiangsu, China), respectively. The mother liquor of CC was prepared by dissolving in absolute ethanol. The concentration of ethanol in the working solution subsequently administered to each treatment group was less than 0.1%. Antibodies against Bcl-2 (B-cell lymphoma-2), Bax (Bcl-2 associated X), PCNA, NF-κB p65, Phospho-NF-κB p65 (p-p65), and GAPDH were obtained from Cell Signaling Technology, Inc. (Danvers, MA, USA). Antibodies against ZBTB7A and HRP-labeled goat anti-rabbit IgG were obtained from Abcam, Inc. (Cambridge, MA, USA) and Beyotime (Shanghai, China), respectively.

***Viability laboratory test and synergy analysis***

 The effects of CC and PTX, either alone or in combination, on the viability of MDA-MB-231 cells were detected using Cell Counting Kit-8 (CCK-8, Dojindo, Rockville, MD, USA) laboratory tests. Cells in the logarithmic phase of growth were resuspended in medium and seeded into 96-well plates at 3×10^3^ cells per well. Twenty-four hours later, the cells were treated with different concentrations of CC (50, 100, 200, 400, 800, 1600, or 3200 μM), PTX (0.08, 0.4, 2, 10, 50, 250, or 1250 μM) and CC+PTX (62.5 μM+0.625 μM, 125 μM+1.25 μM, 250 μM+2.5 μM, 500 μM+5.0 μM, 1000 μM+10 μM, or 2000 μM+20 μM). 24 hr, 48 hr, and 72 hr later, the optical density of each well was examined by a microplate reader at 450 nm. GraphPad Prism 8.0 was used to determine the half-maximal inhibitory concentration (IC_50_) of CC and PTX on MDA-MB-231 cells. Compusyn software (ComboSyn Inc, Paramus, NJ, USA) was used to evaluate the joint efficacy of CC and PTX based on the Chou-Talalay methodology (18). The synergistic (CI<1), antagonistic (CI>1), and additive (CI=1) effects of CC+PTX were quantitatively described by the combination index (CI). According to the results of the synergy analysis, the subsequent experiments included the following four groups: the Control group, CC (250 μM) group, PTX (2.5 μM) group, and CC+PTX (250 μM+2.5 μM) group.

***Colony formation assay***

A total of 250 MDA-MB-231 cells resuspended in the medium were seeded into each well of 6-well plates. Twenty-four hours later, the cells were treated according to their groups. After 6 hr, the cells were cultured in a medium without drugs for 2 weeks. After fixing and staining, the clones were counted under an optical microscope and photographed with a camera. Colony formation rate (%)=(number of colonies/250)×100%.

***Hoechst immunofluorescence staining laboratory test***

To observe apoptosis and morphological changes, we submitted the cells of each group to Hoechst 33258 (Beyotime, Nanjing, China) immunofluorescence staining. Forty-eight hours after drug treatment, the cells were fixed and stained according to the instructions of the kit, and then, the difference in apoptosis and the change in cell morphology between the groups were observed under a fluorescence microscope. Five fields were randomly selected from each group to observe the apoptotic rate (AR) by fluorescence microscopy. AR (%)=number of apoptotic cells/total number of cells×100%.

***Flow cytometry***

To assess the differences in the apoptotic rates among the four groups, we submitted the cells of each group to a specified drug treatment. After 48 hr, the collected cells were double-stained using 5 μl Annexin V-APC and 5 μl 7-AAD for 15 min in the dark. Then, the apoptotic rate was detected by flow cytometry (BD FACSCalibur System, USA). Apoptosis rate (%)=(the number of early apoptotic cells+the number of late apoptotic cells)/total number of cells×100%.

***Western blotting***

After treatment with the indicated drugs for 48 hr, the proteins of each group were extracted in a radioimmunoprecipitation laboratory test (Solarbio, Beijing, China) lysis buffer and phenylmethylsulfonyl fluoride (Solarbio, Beijing, China). A BCA protein assay kit (Blue Skies, Shanghai, China) was used to measure the protein concentration of each group. Protein (30 μg per well) was loaded into gels and separated by 12% sodium dodecyl sulfate-polyacrylamide gel electrophoresis. Then, the separated proteins were transferred to polyvinylidene fluoride membranes (Millipore, Milford, USA) and blocked with a Western blocking solution (Beyotime, Shanghai, China) for 15 min. The membranes were then incubated with primary antibodies for 16 hr (at 4 ^°^C overnight) and HRP-labeled goat anti-rabbit IgG for 1 hr in sequence. Enhanced chemiluminescence (Beyotime, Shanghai, China) substrate and ImageJ software were utilized to determine the relative protein expression. The gray value of the GAPDH band was used as the internal reference, and the ratio of the gray value of the target protein to the internal reference was used as the relative expression level.

***Mammary cancer xenograft models***

Twenty-eight 5-week-old female nude mice (14.8~20.9 g) were procured from the Experimental Animal Center of Guangxi Medical University. All of the animal experiments were conducted strictly in accordance with the related principles of the Ethics Committee of Guangxi Medical University Cancer Hospital. One hundred microliters of PBS containing 6×10^6^ MDA-MB-231 cells were subcutaneously injected into the dorsal side of the right thigh root of each nude mouse. 4 days after the formation of subcutaneous tumors, all nude mice were divided into 4 groups (n=7 per group) according to the random number table method based on the tumor volume of the nude mice. The four groups of nude mice were intraperitoneally injected with the same volume of PBS, CC (100 mg/kg), PTX (10 mg/kg), or the combination of CC+PTX. The procedures were repeated every 2 days. The body mass of the nude mice and the length and width of the xenograft tumors were measured and recorded every 4 days. All nude mice were sacrificed 24 days after subcutaneous transplantation, and then, the weight of the xenograft tumors was precisely measured after careful dissection. Tumor volume was evaluated as follows: Volume (cm^3^)=width^2^ (cm^2^)×length (cm)/2.

***Immunohistochemistry***

The xenograft tumor tissues were paraffin-embedded 24 hr later, and then, 4-μm-thick tissue sections were generated. All sections were dewaxed with xylene and dehydrated with different concentrations of ethanol. Subsequently, tissue sections were blocked with goat serum after thermal antigen retrieval and blocking of endogenous peroxidase with 3% H_2_O_2_. Then the samples were incubated with primary rabbit PCNA, rabbit ZBTB7A, and rabbit NF-κB p65 antibodies overnight at 4 ^°^C. The next day, all sections were incubated with horseradish peroxidase-conjugated secondary antibodies (Beyotime, Shanghai, China). Finally, a color reaction was carried out using 3,3’-diaminobenzidine (Meixin Reagent Co. Ltd., Fuzhou, China). Normal rabbit serum was used as a negative control instead of the primary antibody. The tan or tan granules located in the nucleus are positive cells. Five different fields of view were randomly selected in the slice, and 200 breast cancer cells were selected for counting in each field of view, for a total of 1000 cells, and the proportion of positive cells was calculated. 

***TUNEL and DAPI apoptosis staining***

Tissue sections of the nude mice were heated, dewaxed, and dehydrated. Apoptosis in each group was observed strictly according to the instructions of the Tunel (TdT-mediated dUTP nick end labeling) Apoptosis Staining Kit (Beyotime, Shanghai, China). Next, the nuclei were repeatedly stained with 4’,6-diamidino-2-phenylindole (DAPI). Five random areas under high-power fields of view were selected from each slice to observe the apoptotic rate (AR) by immunofluorescence microscopy. AR (%)=number of apoptotic cells/total number of cells×100%.

***Statistical analysis***

All *in vitro* experiments were repeated at least three times. The measurement data for all the experimental data are presented as x̄±s. One-way analysis of variance was applied to compare differences between groups. SPSS 21.0 was applied to statistically analyze all experimental data. *P<*0.05 was defined as a statistically significant difference.

## Results

***CC and PTX decreased the viability of MDA-MB-231 cells***

**Figure 1:**
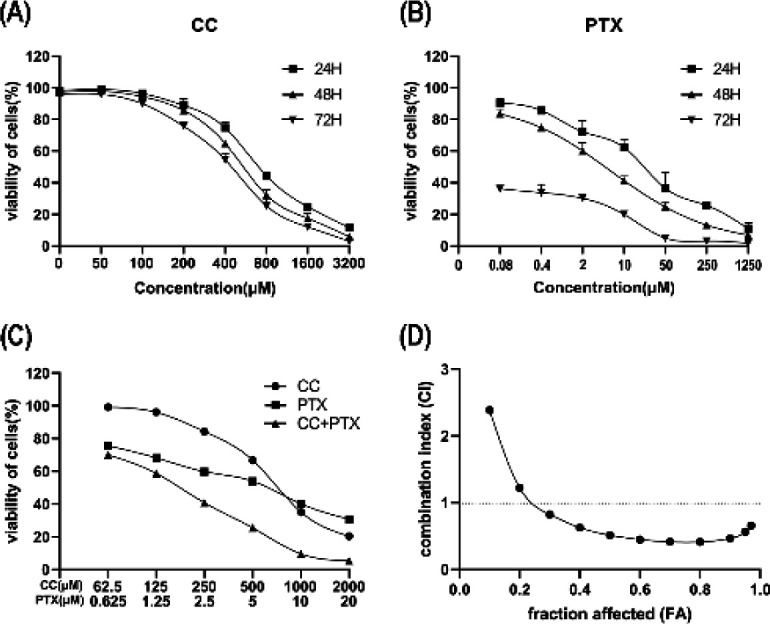
CC and PTX restrained the viability of MDA-MB-231 cells. CC combined with PTX had a synergistic effect on suppressing the viability of MDA-MB-231 cells

A CCK-8 proliferation laboratory test was used to elucidate the effects of CC and PTX on viability of MDA-MB-231 cells. The results demonstrated that CC and PTX decreased the viability of MDA-MB-231 cells in a dose- and time-dependent manner (Figures 1A and B). After MDA-MB-231 cell incubation for 48 hr, the IC_50_ values of CC and PTX were 558.6 μM and 4.4 μM, respectively.

***CC combined with PTX generated a synergistic effect on decreasing the viability of MDA-MB-231 cells***

To verify whether CC combined with PTX decreases the viability of MDA-MB-231 cells, we selected a suitable ratio of CC combined with PTX concentrations according to the IC_50_ of each drug and treated the cells for 48 hr. CC combined with PTX also exerted the same effect on decreasing the viability of MDA-MB-231 cells (Figure 1C). The median effect principle combined with Compusyn software was used to assess the efficacy of various concentrations of CC and PTX on cells. Various combinations of CC+PTX in this study generated synergistic effects on inhibiting the proliferation of MDA-MB-231 cells since the combination index (CI) values were all less than 1 (Table 1). Combined with FA-CI curve analysis (Figure 1D), the CI value reached 0.509 when the affected fraction was 0.593 in the various combinations, and the subsequent experiments included 4 groups: Control group, CC (250 μM) group, PTX (2.5 μM) group, and CC+PTX (250 μM+2.5 μM) group. The above results suggested that CC combined with PTX could synergistically inhibit proliferation of MDA-MB-231 cells.

***CC combined with PTX synergistically inhibited the colony formation of MDA-MB-231 cells***

**Figure 2:**
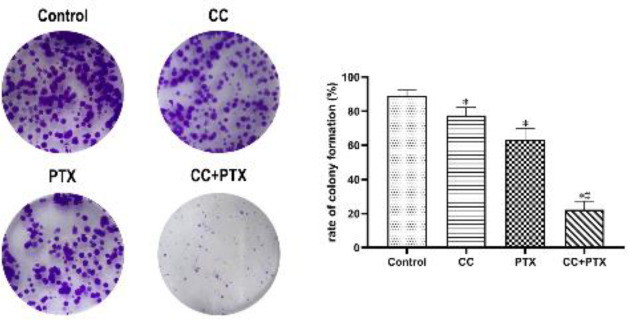
CC combined with PTX restrained colony formation of MDA-MB-231 cells

A colony formation assay was carried out to further validate the effect of CC combined with PTX on inhibiting the proliferation of MDA-MB-231 cells. Data analysis demonstrated that the colony formation rates of CC group, PTX group, and CC+PTX group were obviously lower than that of the Control group, and the CC+PTX group showed the lowest colony formation rate (Figure 2).

***CC combined with PTX generated a synergistic effect on inducing apoptosis of MDA-MB-231 cells in vitro***

**Figure 3:**
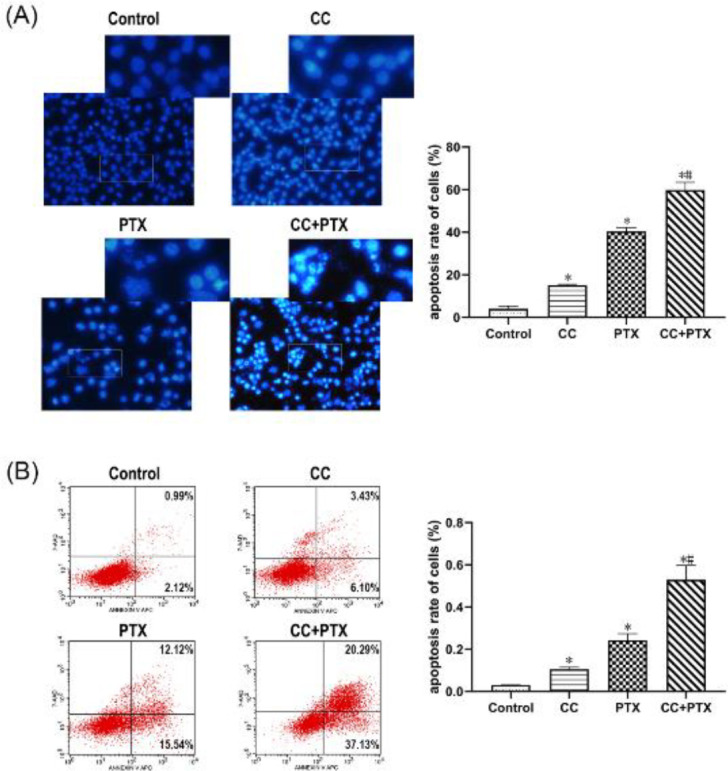
CC combined with PTX promoted apoptosis and morphological changes of MDA-MB-231 cells

To determine whether CC combined with PTX exerted synergistic effects on inducing apoptosis, we submitted the cells of each group to Hoechst 33258 immunofluorescence staining and flow cytometry *in vitro*. After 48 hr of drug treatment, the nuclei of the CC group and PTX group were pyknotic, concentrated, and partially fragmented, and the nuclei of the CC+PTX group were more densely stained and fragmented (Figure 3A). The apoptosis rates of CC group and PTX group were notably greater than that of the Control group, whereas the CC+PTX group showed the highest apoptosis rate (Figure 3A and B).

***CC combined with PTX regulated the protein expression of PCNA, Bcl-2, Bax, ZBTB7A, p-p65, and NF-κB p65 in vitro***

**Figure 4:**
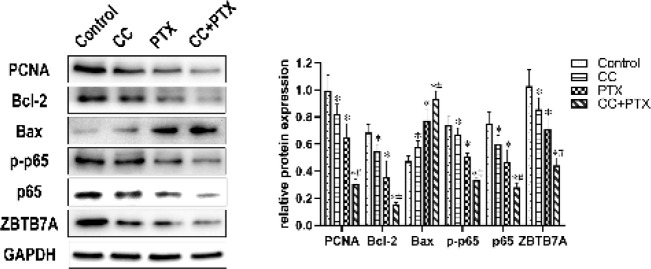
Protein expression of PCNA, Bcl-2, Bax, ZBTB7A, p-p65, and p65 in each group of MDA-MB-231 cells

To elucidate the mechanism underlying the synergy in the CC+PTX group, we submitted the cells of each group to western blotting to detect the protein expression of PCNA, Bcl-2, Bax, ZBTB7A, and the NF-κB signaling pathway-related gene p-p65, p65. After 48 hr of drug treatment, the expression of PCNA, Bcl-2, ZBTB7A, p-p65, and p65 in the CC group and PTX group was dramatically lower than that in the Control group, and the CC+PTX group showed the lowest protein expression (Figure 4). In contrast, the expression of Bax in CC group and PTX group was dramatically higher than that in the Control group, and the CC+PTX group showed the highest expression (Figure 4).

***CC combined with PTX suppressed the growth of xenograft tumors***

**Figure 5:**
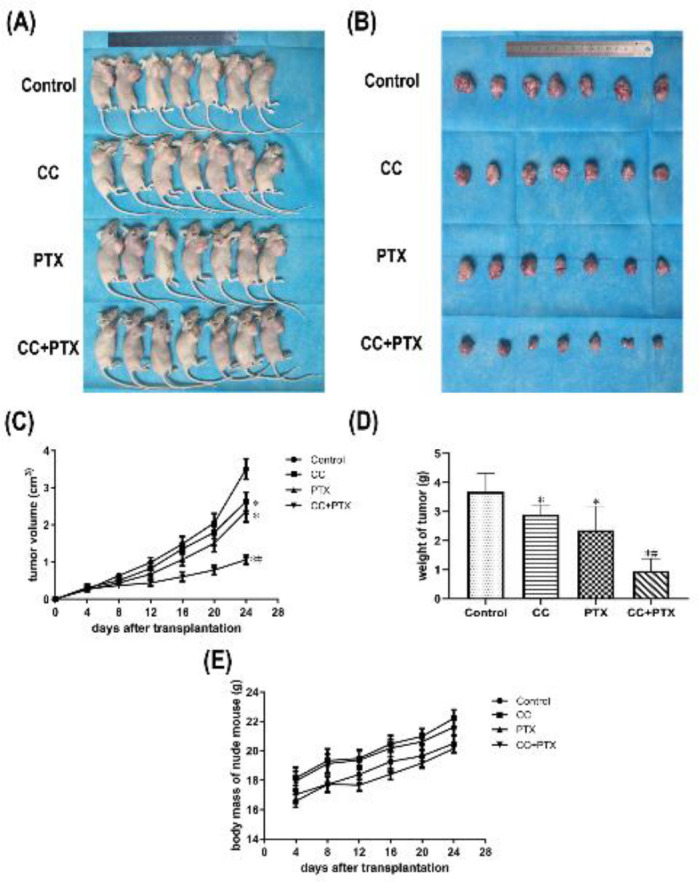
Effects of curcumol combined with paclitaxel on proliferation of MDA-MB-231 cells *in vivo*

To further assess the effect of CC in combination with PTX against mammary cancer *in vivo*, we submitted the cells of each group to establish a nude mouse xenograft model. Compared with that in the Control group, tumor growth (tumor volume and weight) in CC group and PTX group was obviously suppressed, whereas the CC+PTX group showed the most significant suppression (Figure 5A-D). Additionally, there was no obvious difference in the changes in nude mouse body mass before and after treatment between the four groups (Figure 5E).

***CC combined with PTX regulated the expression of PCNA, ZBTB7A, and NF-κB p65 in vivo***

**Figure 6:**
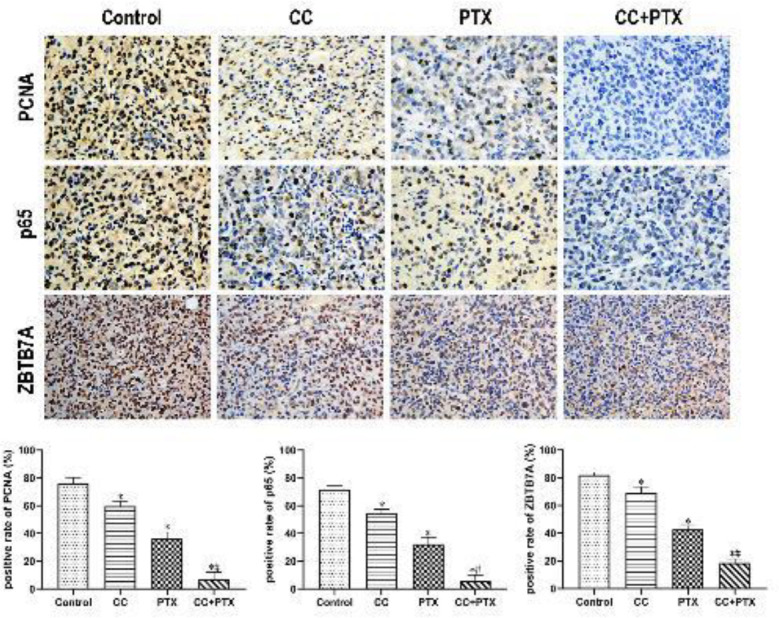
Effects of curcumol combined with paclitaxel on expression of PCNA, ZBTB7A, and p65 in xenograft tumor tissues by immunohistochemistry (×400)

Given the important role of PCNA in initiation of cell proliferation, PCNA is considered a good indicator of the state of tumor cell proliferation. The expression of ZBTB7A can reflect the development and efficacy of mammary cancer to a certain extent. The expression of NF-κB p65 is well correlated with the apoptosis of tumor cells. Therefore, immunohistochemistry was carried out to examine the differences in the proliferation and apoptosis of xenograft cells among the four groups. The protein expression of PCNA, ZBTB7A, and NF-κB p65 was localized to the nucleus, and positive expression was shown by deposition of brown and tan particles in the nucleus. Compared with that in the Control group, the positive expression of PCNA, ZBTB7A, and NF-κB p65 was significantly decreased in CC group and PTX group, whereas the CC+PTX group exhibited the most remarkable decrease (Figure 6).

***CC combined with PTX had a synergistic effect on inducing apoptosis in vivo***

**Figure 7:**
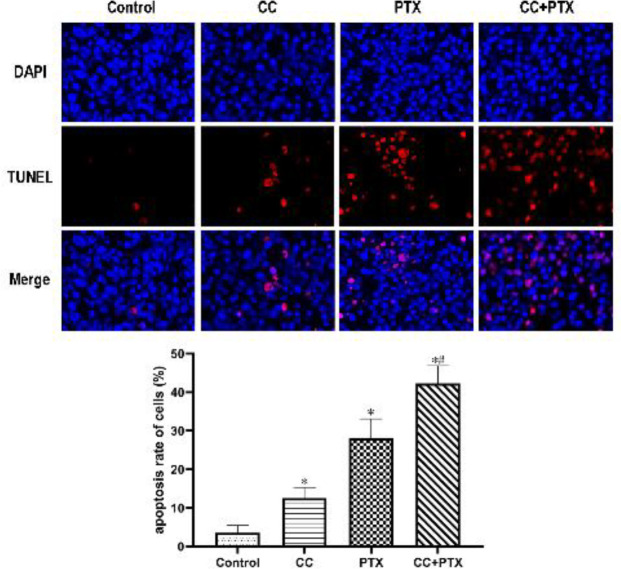
Effects of curcumol combined with paclitaxel on apoptosis of MDA-MB-231 cells in vivo (×200)

TUNEL+DAPI apoptosis staining was used to detect the apoptotic rate of the xenograft tumor tissues in the four groups. The tissue apoptosis rate of CC group and PTX group was remarkably higher than that of the Control group, whereas the apoptosis rate of the PTX+CC group was the highest (Figure 7).

## Discussion

Given the unique biological properties of TNBC and lack of specific and effective targets for treatment, it is still a difficult and hot topic in mammary cancer research. Considering that many patients show chemotherapeutic drug resistance and side effects, a combination of drugs with different anticancer mechanisms characterized by low toxicity and high efficiency is also a new research direction for TNBC treatment. Ling et al. found that the potential mechanism by which CC inhibited the metastasis of MDA-MB-231 mammary cancer cells was AKT- and JNK1/2-mediated NF-κB signaling pathways, and this treatment was accompanied by decreased MMP9 expression (19). Coralyne combined with PTX could promote apoptosis in MDA-MB-231 cells by blocking the cell cycle at the G1 phase and decreasing MMP-9 expression (20). Celecoxib combined with CC suppressed proliferation and promoted apoptosis in non-small cell lung cancer A549 cells by activating the ERK signaling pathway (21). Compared with the two drugs acting alone, the experimental data revealed that CC combined with PTX exhibited dramatically stronger antiproliferative and proapoptotic effects on MDA-MB-231 cells in this study, and the relationship between the two drugs was synergistic.

Many studies have confirmed that the occurrence and progression of malignant tumors are closely associated with the ability of tumor cells to infinitely proliferate and avoid apoptosis (22, 23). PCNA, an ideal cell proliferation indicator, is an intranuclear polypeptide synthesized or expressed in various stages of the cell cycle, such as G1, G2, and S phases, and its expression intensity can accurately predict the proliferative activity and malignancy of various cancer cells (24). Bcl-2 family genes regulate apoptotic processes by affecting the permeability of mitochondria. Bcl-2 and Bax decrease and increase, respectively, the total amount of cytochrome C released from the mitochondria and these effects dramatically influence the apoptotic process of tumor cells (25). Based on analysis of the Bax and Bcl-2 expression data in each experimental group, both CC and PTX could reduce the ratio of Bcl/Bax, and the combination group was the most significantly affected. Thus, the synergistic anticancer effect of CC in combination with PTX against TNBC may be associated with PCNA-mediated proliferation responses and mitochondria-mediated endogenous apoptotic pathways. Whether the combination of the two drugs affects the cell cycle of breast cancer needs to be further diagnosed and confirmed.

NF-κB is an important transcriptional regulator in the nucleus and plays a dual role in anti- or pro-apoptotic processes depending on exposure to different stimuli or specific cell types (26). Mammary cancer tissues and cells overexpress genes related to the NF-κB pathway (27). At present, NF-κB/JNK, NF-κB/TRAF, NF-κB/bcl-2, and NF-κB/TNF axes are common pathways that regulate cell apoptosis and are involved in antitumor activity. There are κB binding sites on the Bcl-2 and Bcl-xl genes. NF-κB can increase the expression of anti-apoptotic genes in the Bcl-2 family, especially Bcl-xl and A1, which exert antiapoptotic effects on reducing the permeability of mitochondrial membranes, inhibiting mitochondrial depolarization, and preventing the release of cytochrome C (28). NF-κB is closely associated with the endoplasmic reticulum stress-mediated JNK signaling pathway (29). In mammary cancer cells, chrysophanol suppresses proliferation and arrests the cell cycle of tumor cells by down-regulating expression of NF-κB p65, p-IκB, and cyclinD1, whereas it promotes PTX-induced apoptosis via the NF-κB/Bcl-2 pathway (10). Our previous research confirmed that ZBTB7A was highly expressed in mammary cancer cells and tissues and that down-regulation of expression of ZBTB7A and NF-κB p65 inhibited the invasion, migration, and metastasis of MDA-MB-231 and MCF-7 cells (30). Our research team has recently performed similar research in this area, and we found that capsaicin inhibits proliferation and induces apoptosis of mammary cancer cells, possibly by inhibiting the ZBTB7A-mediated NF-κB pathway (31). In the present study, the protein expression of ZBTB7A and NF-κB p65 in MDA-MB-231 cells treated with CC and PTX was significantly decreased, and this effect was most obvious in the combination group. These results indicated that CC combined with PTX suppressed the proliferation and promoted the apoptosis of MDA-MB-231 cells *in vitro* and *in vivo*, which may be related to the inhibition of the ZBTB7A and NF-κB signaling pathways. However, further experimental studies involving the addition of NF-κB signaling pathway inhibitors are needed to confirm the above results. In addition, different blood concentrations will significantly affect the proliferation ability of tumor cells, so follow-up *in vitro* experiments need to closely monitor blood concentrations of both CC and PTX and evaluate the optimal treatment cycle.

(A) Effects of CC on viability of MDA-MB-231 cells at different time points. (B) Effects of PTX on viability of MDA-MB-231 cells at different time points. (C) Effects of CC, PTX, and CC+PTX on viability of MDA-MB-231 cells for 48 hr. (D) FA-CI curve of CC combined with PTX on MDA-MB-231 cells

CC: Curcumol; PTX: Paclitaxel; FA: Fraction affected; CI: Combination index

**Table 1:** Synergistic effect analysis of CA combined with PTX in MDA-MB-231 cells

	PTX	CI	FA
62.5	0.625	0.694	0.300
125	1.25	0.657	0.413
**2** **50**	2.5	0.509	0.593
**500**	5	0.493	0.746
**1000**		0.396	0.905
**2000**		0.533	0.947

CC: Curcumol; PTX: Paclitaxel; CI: Combination index; FA: Fraction of cell viability affected

Control, cells treated with 0.1% absolute ethanol; CC, cells treated with curcumol (250 μM); PTX, cells treated with paclitaxel (2.5 μM); CC+PTX, cells treated with curcumol and paclitaxel (250 μM+2.5 μM); N=3, *P<0.05 versus Control group; #*P<*0.05 versus CC and PTX group

(A) Effect of each group on apoptosis and morphological changes of MDA-MB-231 cells was detected by Hoechst 33258 immunofluorescence staining (×200). (B) Effect of each group on apoptosis of MDA-MB-231 cells detected by flow cytometry. N=3, *P<0.05 versus Control group; #*P<*0.05 versus CC and PTX group

N=3, *P<0.05 versus Control group; #*P<*0.05 versus CC and PTX group

(A) A general view of xenograft nude mice. (B) Anatomical view of xenograft tumors. (C) Growth curve of xenograft tumors. (D) Tumor weight of each group. (E) Trend of body mass changes in each group of nude mice (*P>*0.05). N=7, *P<0.05 versus Control group; #*P<*0.05 versus CC and PTX group

N=7, *P<0.05 versus Control group; #*P<*0.05 versus CC and PTX group

N=7, *P<0.05 versus Control group; #*P<*0.05 versus CC and PTX group

## Conclusion

CC combined with PTX exerts synergistic antiproliferative and proapoptotic effects on MDA-MB-231 cells. The combination of CC and PTX is a novel strategy for the treatment of triple-negative breast cancer (TNBC). The underlying mechanism may be related to down-regulation of ZBTB7A expression via the NF-κB signaling pathway.

## Authors’ Contributions

AM and CW designed the entire experimental route. CW and QQ guided implementation. AM conducted the experiment, analyzed data, and drafted the manuscript. WY provided literature search assistance. CW and QQ provided fund support.

## Conflicts of Interest

The authors report no conflicts of interest in this work.
